# The lysosomal V-ATPase a3 subunit is involved in localization of Mon1-Ccz1, the GEF for Rab7, to secretory lysosomes in osteoclasts

**DOI:** 10.1038/s41598-022-12397-w

**Published:** 2022-05-19

**Authors:** Naomi Matsumoto, Mizuki Sekiya, Ge-Hong Sun-Wada, Yoh Wada, Mayumi Nakanishi-Matsui

**Affiliations:** 1 Division of Biochemistry, School of Pharmacy, Iwate Medical University, Idaidori 1-1-1, Shiwa, Yahaba, Iwate 028-3694 Japan; 2 Department of Biochemistry, Faculty of Pharmaceutical Sciences, Doshisha Women’s College, Kyotanabe, Kyoto 610-0395 Japan; 3 Division of Biological Sciences, Institute of Scientific and Industrial Research, Osaka University, Ibaraki, Osaka 567-0047 Japan

**Keywords:** Membrane trafficking, Organelles

## Abstract

We have shown previously that the lysosomal a3 isoform of the a subunit of vacuolar-type ATPase (V-ATPase) interacts with inactive (GDP-bound form) Rab7, a small GTPase that regulates late endosome/lysosome trafficking, and that a3 recruits Rab7 to secretory lysosomes in mouse osteoclasts. This is essential for outward trafficking of secretory lysosomes and thus for bone resorption. However, the molecular mechanism underlying the recruitment of Rab7 by a3 remains to be fully elucidated. Here, we showed that a3 interacts with the Mon1A-Ccz1 complex, a guanine nucleotide exchange factor (GEF) for Rab7, using HEK293T cells. The interaction was mediated by the amino-terminal half domain of a3 and the longin motifs of Mon1A and Ccz1. Exogenous expression of the GEF promoted the interaction between a3 and Rab7. Mon1A mutants that interact inefficiently with Rab7 interacted with a3 at a similar level to wild-type Mon1A. Lysosomal localization of endogenous Ccz1 was abolished in osteoclasts lacking a3. These results suggest that the lysosomal a3 isoform of V-ATPase interacts with Mon1A-Ccz1, and that a3 is important for Mon1A-Ccz1 localization to secretory lysosomes, which mediates Rab7 recruitment to the organelle.

## Introduction

Osteoclasts tightly attach to bone and secrete lysosomal enzymes into the bone resorption lacuna, an interspace between the cell and bone, to digest bone matrix^1,2^. Secretory lysosomes in osteoclasts move toward and fuse with the plasma membrane to secrete enzymes. During this secretory process, a lysosomal proton pump V-ATPase is co-transported to the plasma membrane, where the pump acidifies the bone resorption lacunae to establish adequate acidic conditions for lysosomal enzymes, as well as to dissolve calcium phosphate in bone^3–6^. Although the secretory lysosomes are essential for bone resorption, the mechanism underlying the anterograde trafficking of lysosomes from the perinuclear region to the cell surface has not been fully elucidated.

Rab7 is a member of the Rab small GTPase family. It is involved in microtubule-dependent transport of late endosomes and lysosomes. In osteoclasts, Rab7 regulates outward trafficking of secretory lysosomes^4–8^. The activity of Rab proteins is regulated by cycling between GTP- and GDP-bound forms. The guanine nucleotide exchange factors (GEFs) activate the corresponding small GTPases by exchanging the bound guanine nucleotides^8–10^. After loading of GTP by GEFs, Rab proteins stably localize to a specific organelle membrane, where they form a large machinery that regulates organelle trafficking along the cytoskeleton^9,10^. Although the functions of Rab small GTPases in organelle trafficking have been described, the molecular mechanisms underlying the recruitment of Rab proteins to target organelles remain largely unknown.

The Mon1-Ccz1 complex is the GEF for Rab7^11–13^. Mon1 and Ccz1 interact through their longin domains to form a heterodimer that interacts with GDP-bound Rab7 and is involved in GEF activity^12,14^. The GEF is required for Rab7 localization to the target organelle in yeast^14^.

**Figure 1:**
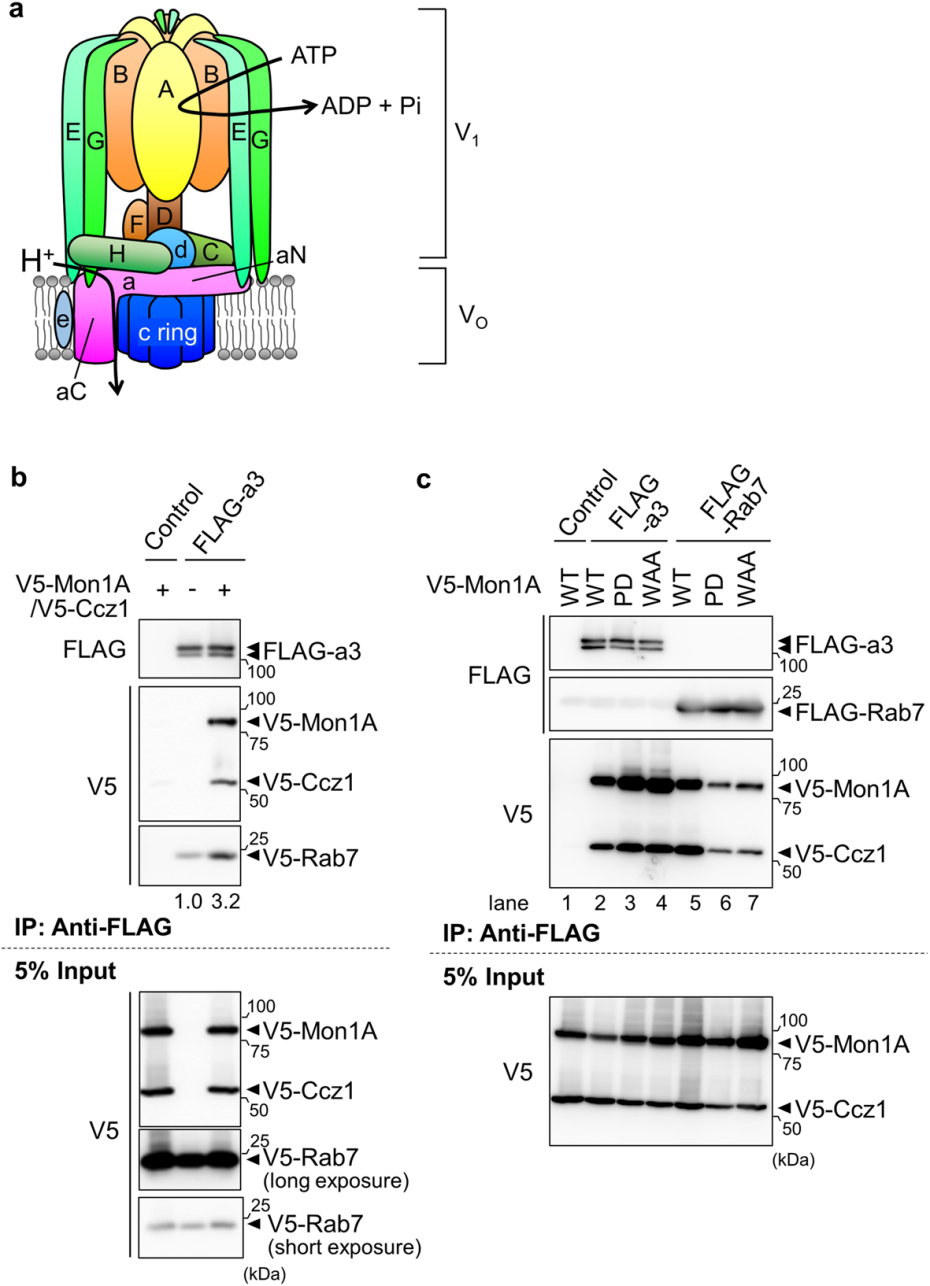
Interaction of the V-ATPase a3 subunit with Rab7 and Mon1A-Ccz1. (**a**) Schematic illustration of the subunits of the mammalian proton pump V-ATPase. aN and aC represent the cytosolic amino-terminal half domain and membrane-intrinsic carboxy-terminal half domain of the a subunit, respectively. (**b**) Interaction between a3, Rab7, Mon1A, and Ccz1. FLAG-tagged a3, V5-tagged GDP-fixed form of Rab7(T22N), V5-tagged Mon1A, and V5-tagged Ccz1 were co-expressed in HEK293T cells. The cell lysates were immunoprecipitated with anti-FLAG antibody. The precipitates were analyzed using antibodies against FLAG and V5. Control indicates experiments using cells transfected with an empty vector instead of the FLAG-a3 expression plasmid (Control). Five percent of the cell lysate used for immunoprecipitation was also subjected to western blotting (5% Input). Numbers below the blots represent relative signal intensities of V5-Rab7. (**c**) Interaction of Mon1A mutants with a3 or Rab7. FLAG-a3 or FLAG-Rab7(T22N) was co-expressed with the V5-Mon1A mutant and V5-Ccz1. Immunoprecipitation was performed as described in (**b**). PD and WAA indicate Mon1A mutants with amino acid replacements G166P/K167D and N262W/D264A/R266A, respectively. Unprocessed scans of immunoblots in (**b**) and (**c**) are shown in Supplementary Fig. S3 online.

V-ATPase is a proton pump that catalyzes the transport of protons across the membrane using the energy from ATP hydrolysis^15–19^. V-ATPase is composed of V_1_ and V_o_ domains; V_1_, the catalytic domain, is formed by a complex of A_3_B_3_CDE_3_FG_3_H subunits, whereas V_o_ is a proton pathway formed by a complex of ac_9_c”de subunits (Fig. 1a). Six subunits of V-ATPase have tissue- or cell-specific isoforms; for example, the a subunit has 4 isoforms, a1, a2, a3, and a4. The a4 isoform is selectively expressed in transporting epithelia of several tissues, including kidney, epididymis, retina, and inner ear, whereas the other a1-a3 isoforms are expressed ubiquitously. The a1, a2, and a3 are localized in coated vesicles, Golgi apparatus/early endosomes, and late endosomes/lysosomes, respectively ^15–19^.

We previously demonstrated that a3 binds to the GDP-bound form of Rab7 but not to GTP-loaded Rab7, and that a3 is responsible for recruitment of Rab7 to secretory lysosomes; this leads to trafficking of organelles toward the cell periphery and thus the delivery of bone-resorbing enzymes to the resorption lacunae along with the proton pump^6,19^. This finding, together with those of previous studies, suggest that V-ATPase has a dual function in osteoclasts: the acidification of bone resorption lacunae and trafficking of secretory lysosomes^6,18,19^.

In this study, we suggested that the V-ATPase a3 subunit plays an important role in localization of the Mon1A-Ccz1 GEF complex to secretory lysosomes via interaction with the GEF. Mon1-Ccz1 recruit Rab7 to their resident organelle, where it is converted to the GTP-loaded form to trigger organelle trafficking^11–14,20–24^. Therefore, Mon1A-Ccz1 recruitment to the specific organelle is the essential first step for organelle trafficking in osteoclasts. We suggest here that the molecular interaction between a3 and the GEF is the mechanistic link for the recruitment of the GDP-bound Rab7 to secretory lysosomes and subsequent activation of Rab7.

## Results

### The a3 subunit interacts with the Mon1A-Ccz1 complex

We previously showed that the a3 subunit of V-ATPase specifically interacts with the GDP-bound form of Rab7^6^. The nucleotide cycling of Rab proteins from GDP- to GTP-bound forms requires the function of GEFs, which physically interact with GDP-bound small GTPases^20,25^. Therefore, we presumed that a3 and Rab7 would form a complex with the Rab7 GEF, Mon1A-Ccz1. To examine this, we co-expressed FLAG-tagged a3, V5-tagged Mon1A, V5-tagged Ccz1, and V5-tagged GDP-fixed form of Rab7 carrying the mutation T22N in HEK293T cells. Anti-FLAG antibody precipitated the V5-tagged Mon1A and V5-tagged Ccz1 (Fig. 1b, V5), indicating that a3 interacted with the Mon1A-Ccz1 complex. The GDP-fixed Rab7 was also precipitated with a3 (Fig. 1b, V5, V5-Rab7). The amount of Rab7 in the complex of a3 increased threefold when V5-Mon1A and V5-Ccz1 were exogenously expressed, suggesting that the complex was assembled with a certain stoichiometry in which the amounts of Mon1A and Ccz1 were the limiting factor for complex formation.

To test whether a3 interacts with Mon1A-Ccz1 in a Rab7-dependent manner, we expressed FLAG-a3 and V5-Mon1A carrying the G166P/K167D (PD) and N262W/D264A/R266A (WAA) mutations, which preserve the formation of the Mon1A-Ccz1 complex but disrupt its interaction with Rab7^12,14^, and performed immunoprecipitation. Both the PD and WAA mutants interacted with FLAG-GDP-fixed Rab7 less efficiently than did wild-type (WT) Mon1A (Fig. 1c, V5, lanes 5–7), confirming that these mutations affected its binding to Rab7. By contrast, FLAG-a3 still associated with V5-Mon1A mutants (Fig. 1c, V5, lanes 2–4), indicating that the interaction between V-ATPase a3 and the GEF did not require the physical association between Rab7 and the complex.

### The a3 subunit is required for Ccz1 localization to secretory lysosomes

**Figure 2:**
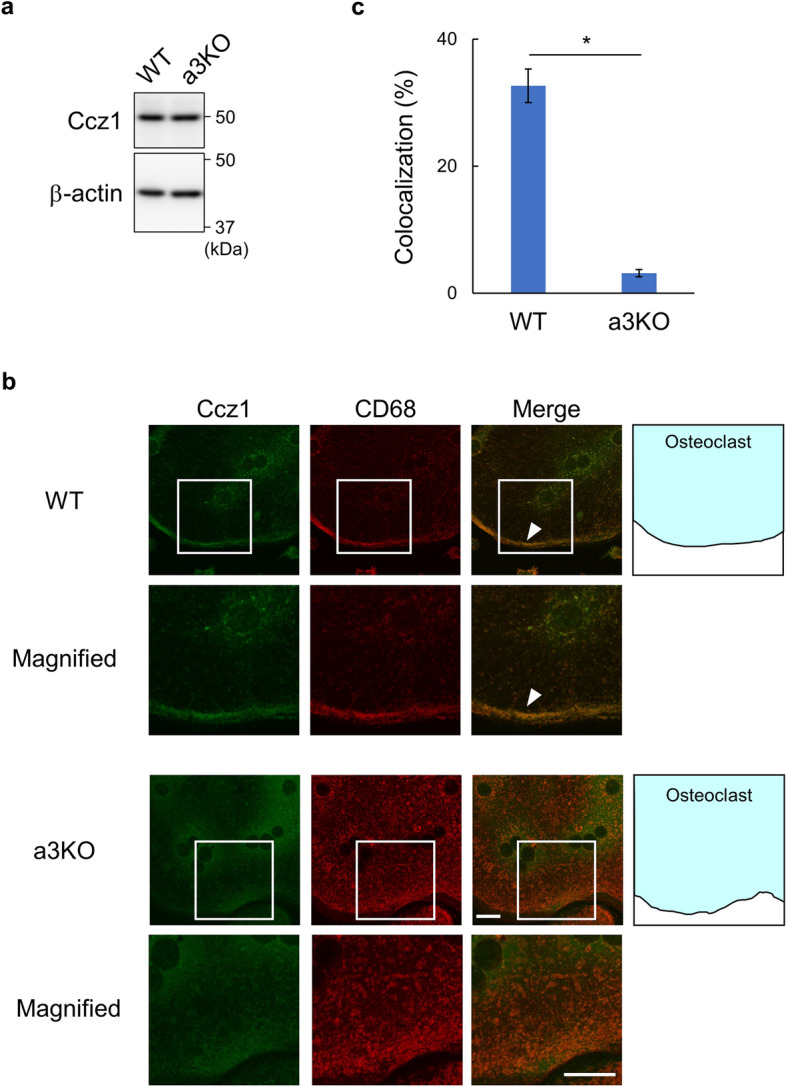
Localization of Ccz1 in WT and a3KO osteoclasts. (**a**) Ccz1 expression in WT and a3KO osteoclasts. Osteoclasts were differentiated from spleen macrophages derived from WT and a3KO mice, and the corresponding cell lysates were subjected to western blotting using anti-Ccz1 and anti-β-actin antibodies. Unprocessed scans of immunoblots are shown in Supplementary Fig. S4 online. (**b**) Localization of endogenous Ccz1 and lysosomes. WT and a3KO osteoclasts were obtained as in (**a**) and stained with anti-Ccz1 (green) and anti-CD68 (red) antibodies. Arrowheads indicate co-localization of Ccz1 and CD68 at the cell periphery. Schematic illustrations of osteoclasts and magnified images are also shown. The bars indicate 20 µm. (**c**) Lysosomal localization of Ccz1. Co-localization of Ccz1 and CD68 was observed, and the ratios of CD68-positive pixels that were also Ccz1-positive were calculated. Fifteen cells were randomly selected from three experiments using WT and a3KO cells. Means are shown with SEM. *P < 0.001 (unpaired two-tailed Student’s *t*-test).

Rab7 plays an essential role in the trafficking of secretory lysosomes in osteoclasts^4–8^. We previously demonstrated that the V-ATPase a3 subunit is required for recruitment of Rab7 to secretory lysosomes^6^. A pertinent question, therefore, was whether a3 is required for lysosomal localization of the GEF. WT and a3-knockout (a3KO) osteoclasts were prepared from spleen macrophages. The expression levels of Ccz1 in a3KO osteoclasts were similar to those in WT cells (Fig. 2a). To test the specificity of the anti-Ccz1 antibody, Ccz1 expression was suppressed in spleen macrophages by shRNA knockdown, and immunostaining was performed with the anti-Ccz1 antibody. Ccz1 signals were rarely observed in Ccz1-knocked down cells, but were clearly visible in control cells, as shown in Supplementary Fig. S1, indicating that the anti-Ccz1 antibody specifically detects Ccz1.

Numerous CD68-positive secretory lysosomes accumulate in the peripheral region of WT osteoclasts; however, this peripheral localization was not observed in a3KO cells (Fig. 2b, CD68). Ccz1 showed a similar peripheral localization along with CD68 in WT cells (Fig. 2b, arrowheads). Ccz1 also localized to the internal organelles with a more central location, which was also positive for the lysosomal marker protein CD68. By contrast, in a3KO cells, Ccz1 did not accumulate on any organelle membrane and was detected as diffuse signals throughout the cytosol. Morphometric analyses confirmed that the co-localization of Ccz1 and CD68 was significantly reduced to nearly 3% in a3KO cells (Fig. 2c). These results suggest that the V-ATPase a3 isoform is required for the localization of Ccz1 to secretory lysosomes.

### Identification of a3 domains involved in the interaction with Mon1A-Ccz1

**Figure 3:**
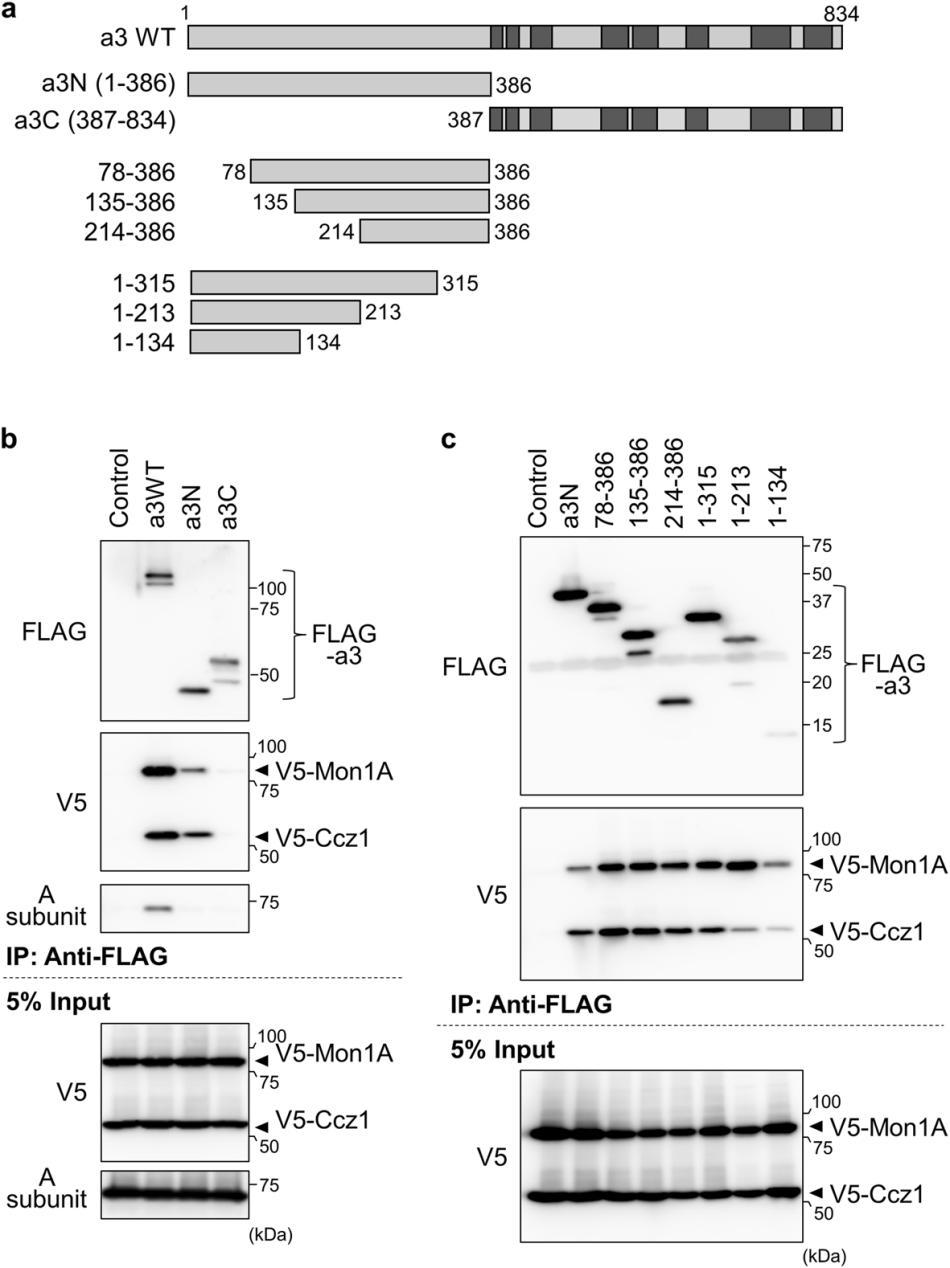
Identification of the a3 domain involved in the interaction with Mon1A-Ccz1. (**a**) Structures of a3 deletion mutants. The structures of a3 deletion mutants are schematically illustrated. a3N and a3C represent the cytosolic amino-terminal half of a3 and the carboxy-terminal half with eight transmembrane domains (dark gray), respectively. Numbers indicate amino acids from the amino terminus. (**b**, **c**) Interaction between a3 deletion mutants and Mon1A-Ccz1. FLAG-tagged halves of a3 (**b**) or FLAG-tagged deletion mutants of a3N (**c**) were co-expressed with V5-Mon1A and V5-Ccz1 in HEK293T cells. Immunoprecipitation using anti-FLAG antibody and western blotting were performed as described in Fig. 1. FLAG-a3 derivatives were not expressed in control experiments (Control). Unprocessed scans of immunoblots in (**b**) and (**c**) are shown in Supplementary Fig. S5 online.

Next, we determined which domains are important for the interaction between a3 and Mon1A-Ccz1. The a3 subunit is a large protein composed of more than 800 amino acids, and it has eight transmembrane segments in the carboxy-terminal half (a3C) (amino acids 387–834) that form a proton pathway with the c-ring (Figs. 1a and 3a)^18^. Its amino-terminal half (a3N) (amino acids 1–386) faces the cytosol and interacts with other subunits of the V_1_ sector (Fig. 1a). FLAG-tagged versions of a3N, a3C, or a3WT (full-length) were expressed in HEK293T cells transfected with V5-Mon1A and V5-Ccz1, and their interaction was examined by immunoprecipitation. FLAG-a3N and FLAG-a3WT formed a complex with V5-Mon1A and V5-Ccz1, whereas FLAG-a3C failed to pull down the GEF complex (Fig. 3b, V5). This indicated that a3 interacted with Mon1A-Ccz1 mainly through its amino-terminal half domain. Neither a3N nor a3C interacted with the V-ATPase A subunit of the V_1_ domain (Fig. 3b, A subunit).

We then prepared a series of FLAG-a3N mutants with further deletions from the amino terminus or from the carboxy terminus (Fig. 3a), and examined their interaction with V5-Mon1A and V5-Ccz1. Similar to a3N, these mutants exhibited similar or more efficient interaction with V5-Mon1A and V5-Ccz1 (Fig. 3c, V5), indicating that each part of a3N is involved in the interaction with Mon1A-Ccz1. Interestingly, a3N deletion mutant 1–213 interacted weakly with Ccz1, but strongly with Mon1A (Fig. 3c, V5). This suggests that Mon1A and Ccz1 bind to different regions in a3N. Ccz1 showed lower affinity for mutant 1–213 than for mutant 1–315, suggesting that Ccz1 mainly interacts with the region between amino acids 214 and 315 of a3N. On the other hand, Mon1A showed lower affinity for mutant 1–134 than for mutant 1–213, suggesting that the 135–213 region of a3N is important for interaction with Mon1A. Unexpectedly, Mon1A also interacted with mutant 214–386. Thus, Mon1A appears to interact with a relatively wide region in the 135–386 amino acid region of a3N.

### Identification of Mon1A and Ccz1 domains involved in the interaction with a3N

**Figure 4:**
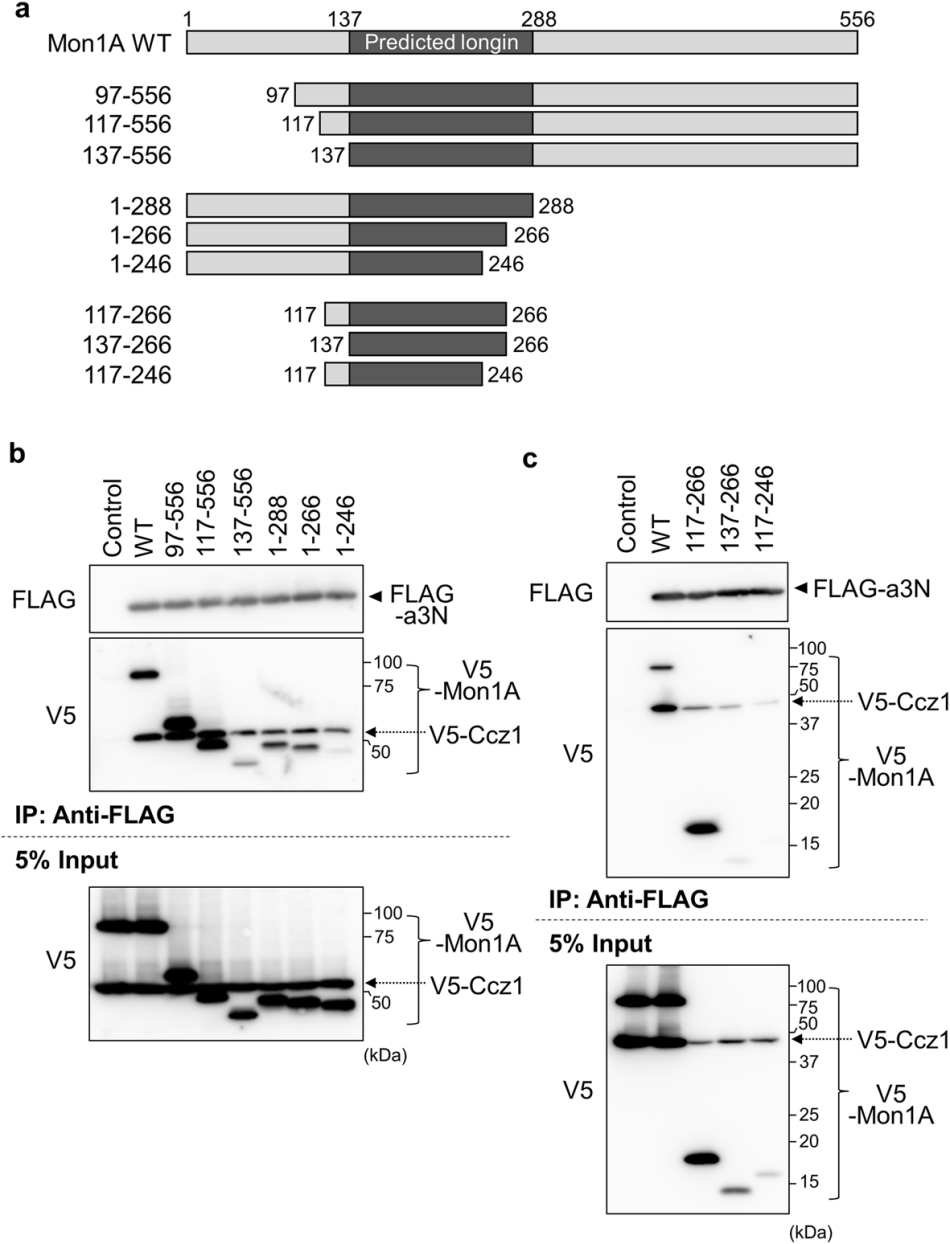
Identification of the Mon1A domain involved in the interaction with a3N. (**a**) The structures of Mon1A deletion mutants are schematically illustrated. The predicted longin domain is indicated in dark gray. Numbers indicate amino acids from the amino terminus. (**b**, **c**) Interactions between Mon1A deletion mutants, a3N, and Ccz1. V5-tagged deletion mutants of Mon1A were co-expressed with FLAG-a3N and V5-Ccz1 in HEK293T cells, and immunoprecipitation was performed using anti-FLAG antibody as described in Fig. 1. FLAG-a3N was not expressed in control experiments (Control). Unprocessed scans of immunoblots in (**b**) and (**c**) are shown in Supplementary Fig. S6 online.

Mouse Mon1A and Ccz1 are proteins composed of 556 and 480 amino acid residues, respectively. We created a series of Mon1A mutants containing deletions in the amino-terminal or the carboxy-terminal region (Fig. 4a). FLAG-a3N, V5-tagged versions of these mutants, and V5-tagged Ccz1 were expressed in HEK293T cells, and molecular interactions were examined by immunoprecipitation using anti-FLAG antibody. Significant amounts of Mon1A mutants with the 97–556 and 117–556 segments co-immunoprecipitated with a3N, whereas only a small amount of mutant 137–556 was recovered (Fig. 4b, V5). This indicated that the 117–556 amino acid region contained (an) important domain(s) for the interaction. Among the carboxy-terminal deletion mutants, mutant 1–288 and mutant 1–266 exhibited a significant interaction with a3N compared with the 1–246 mutant (Fig. 4b, V5), indicating that 1–266 contained (an) important region(s) for the interaction. Consistent with these results, mutant 117–266 showed significant interaction with a3N (Fig. 4c, V5). Our attempt to narrow further failed because mutants containing short segments (137–266 and 117–246) were hardly expressed in the cells (Fig. 4c, 5% Input). Nonetheless, we identified amino acids 117–266 in Mon1A as a region important for interaction with a3N. This region overlapped with the longin motif found in fungal proteins, which is implicated in heterodimerization, interaction with GDP-bound Rab7, and GEF activity of the Mon1-Ccz1 complex^12,14^. Mon1A deletion mutant 1–246 hardly interacted with a3N, while Ccz1 still bound to a3N (Fig. 4b, V5). Since HEK293T cells express endogenous Mon1, Ccz1 likely formed a heterodimer with endogenous Mon1 to interact with a3N in cells expressing the Mon1A deletion mutant. Another possibility is that Ccz1 may interact with a3N independently of Mon1A.

**Figure 5:**
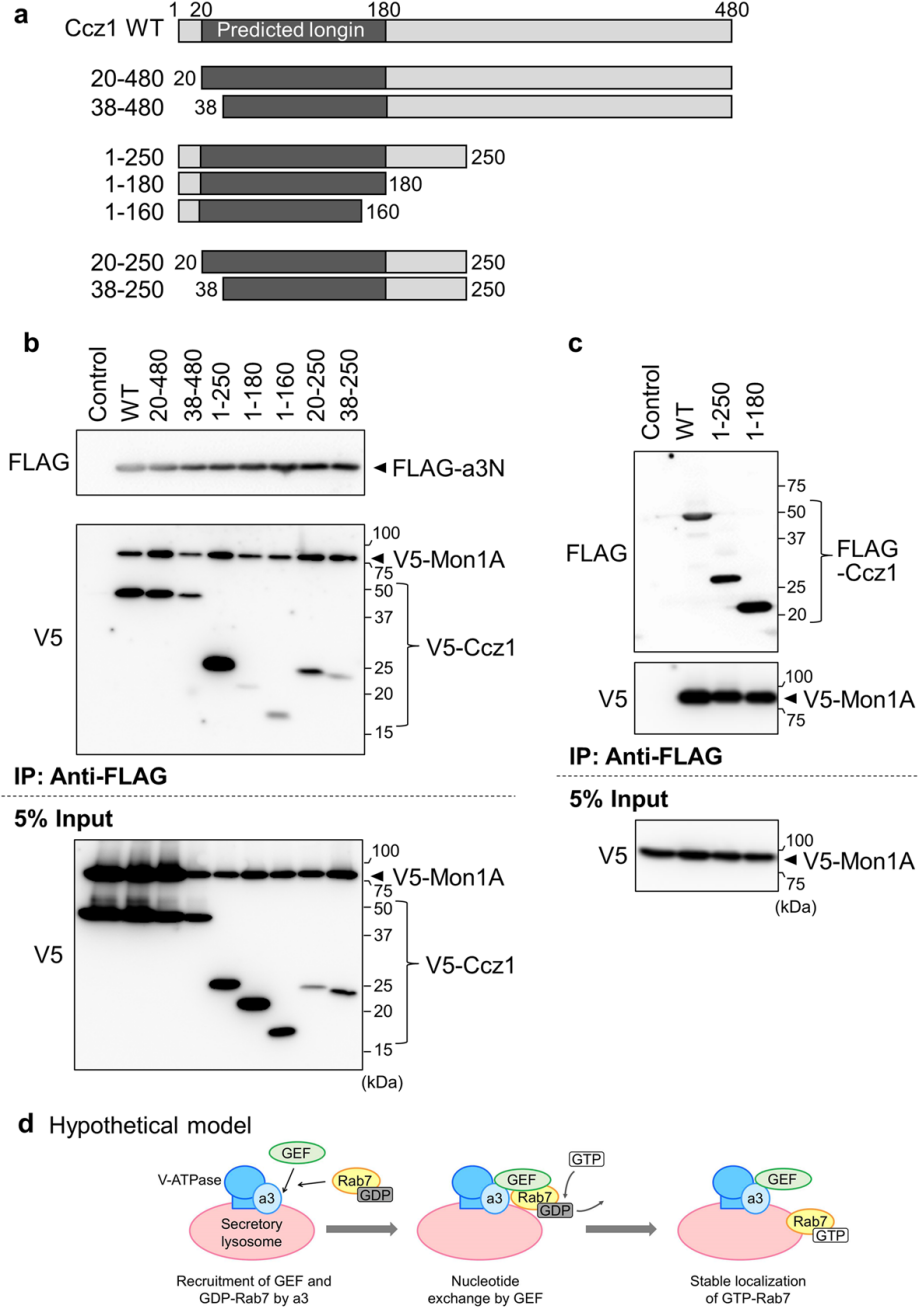
Identification of the Ccz1 domain involved in the interaction with a3N. (**a**) The structures of Ccz1 deletion mutants are schematically illustrated. The predicted longin domain is indicated in dark gray. (**b**) Interactions between Ccz1 deletion mutants, a3N, and Mon1A. V5-tagged deletion mutants of Ccz1 were co-expressed with FLAG-a3N and V5-Mon1A in HEK293T cells, and immunoprecipitation using anti-FLAG antibody was performed as described in Fig. 1. FLAG-a3N was not expressed in a control experiment (Control). (**c**) Interaction of the predicted Ccz1 longin domain with Mon1A. FLAG-tagged deletion mutants of Ccz1 were co-expressed with V5-Mon1A in HEK293T cells, and immunoprecipitation using anti-FLAG antibody was performed as described in Fig. 1. FLAG-Ccz1 was not expressed in a control experiment (Control). Unprocessed scans of immunoblots in (**b**) and (**c**) are shown in Supplementary Figs. S7 and S8 online. (**d**) Hypothetical model of Rab7 recruitment to osteoclast secretory lysosomes and its activation by the Mon1-Ccz1 GEF. The a3 isoform of V-ATPase recruits GDP-bound Rab7 to secretory lysosomes through interaction with the Rab7 GEF, Mon1-Ccz1. After guanine nucleotide exchange catalyzed by the GEF, activated GTP-loaded Rab7 stably localizes to the secretory lysosome membrane and forms a large complex on lysosomes to trigger anterograde trafficking.

We next examined the interaction between a3N and the Ccz1 mutants (Fig. 5a). V5-Ccz1 mutant 20–480 and mutant 38–480 co-immunoprecipitated with FLAG-a3N (Fig. 5b, V5), and mutant 1–250 also interacted with a3N. We then prepared mutant 20–250 and mutant 38–250. These are mutants with deletions from both termini. Although these mutants showed lower expression levels in HEK293T cells than did other mutants (Fig. 5b, 5% Input), a strong signal was obtained for mutant 20–250 compared with mutant 38–250 (Fig. 5b, V5), indicating that amino acids 20–250 of Ccz1 are an important region for interaction with a3N.

The PSIPRED workbench showed that this important region (20–250) of mouse Ccz1 forms a longin-like fold, a secondary structure composed of five antiparallel β-sheets and three α-helices (β1–β2–α1–β3–β4–β5–α2–α3) commonly found in longin domains^26^. We then confirmed whether this region is necessary for the interaction with Mon1A. To confirm the interaction between Mon1A and the amino-terminal half of Ccz1, V5-Mon1A and a FLAG-Ccz1 mutant of segment 1–250 or 1–180 were expressed in HEK293T cells, and immunoprecipitation was performed using anti-FLAG antibody. Both mutants clearly pulled down V5-Mon1A (Fig. 5c, V5), suggesting that Ccz1 interacts with Mon1A via the longin motif in the amino-terminal region of Ccz1. However, for interaction between a3N and Ccz1, the 20–180 region is not sufficient, and the 181–250 region is also required. Taken together, these observations suggest that the extra region comprises an interface for a3N.

In addition, we examined whether the regions important for interaction with a3N were also important for heterodimer formation. Mon1A deletion mutant 1–246 interacted more weakly with a3N than mutant 1–266 (Fig. 4b, V5), and Ccz1 deletion mutant 38–250 interacted more weakly with a3N than mutant 20–250 (Fig. 5b, V5). The deletion mutants showing a weaker interaction with a3N also exhibited inefficient heterodimer formation (Supplementary Fig. S2, compare co-precipitated V5-Mon1A signals with corresponding signals in 5% input), indicating that the regions important for the interaction with a3N were also important for interaction between Mon1A and Ccz1. Since the predicted longin domains are partly truncated in these deletion mutants (Figs. 4a and 5a), the results are consistent with the previous observation that the longin domains in Mon1A and Ccz1 are important for heterodimer formation^11^.

## Discussion

We previously demonstrated that the a3 subunit of the proton pump V-ATPase binds to and recruits Rab7 to secretory lysosomes in osteoclasts^6^. This molecular interaction between the lysosomal proton pump and the Rab protein is important for the anterograde trafficking of secretory lysosomes and thus supports bone resorption. The present results suggest that a3 interacts with the Rab7 GEF, Mon1A-Ccz1, and plays an important role in recruitment of the GEF to secretory lysosomes in osteoclasts. However, to elucidate that Mon1A-Ccz1 interaction with a3 is essential for their recruitment to secretory lysosomes, it will be important to show that Mon1A and Ccz1 mutants that do not interact with a3 do not localize to secretory lysosomes in osteoclasts.

The interaction between Mon1A-Ccz1 and a3 does not require the association of Rab7 with the GEF complex, suggesting that the a3-GEF complex has regulatory roles upstream of Rab7 recruitment. This scenario is consistent with the observation that the GEF complex increased the co-immunoprecipitation of Rab7 with a3. Based on these observations, we suggest that Mon1A-Ccz1, together with the V-ATPase on secretory lysosome membranes, recruits Rab7 to the organelle (Fig. 5d). Consistent with this, Mon1-Ccz1 is required for vacuolar localization of the yeast Rab7, Ypt7^14^. After activation by the GEF, Rab7 appears to localize stably on lysosomal membranes, where it assembles a large molecular machinery for attaching to and moving along microtubules^27–29^. To elucidate the precise mechanism, it will be important to determine the tertiary structure of the complex between a3 and Mon1A-Ccz1 using purified recombinant proteins.

In this study, we suggested that the lysosomal V-ATPase plays an important role in the trafficking of secretory lysosomes via its molecular interaction with the trafficking machineries. The mechanistic link between the V-ATPase, the GEF for Rab7, and Rab7 appears to enable not only precise membrane localization of the trafficking machinery, but also site-specific activation of the Rab7 protein.

The anterograde trafficking of secretory lysosomes is essential for bone resorption. Therefore, the present findings are important to understand the molecular basis of bone homeostasis and may contribute to the development of novel clinical strategies for the treatment of pathogenic disorders, including osteopetrosis and osteoporosis. Secretory lysosomes are not restricted to osteoclasts, and are also involved in perforin secretion in cytotoxic T cells and melanin secretion in melanocytes^30,31^. Therefore, it is of interest to examine whether a similar mechanism is involved in trafficking of secretory lysosomes in non-osteoclast cells.

Although more than 60 members of the Rab family have been identified in mammals and their role in organelle trafficking has been established, how each Rab protein is recruited to specific organelles remains a long-standing question^20,21^. This study suggested, at least partly, the molecular mechanism underlying Rab protein recruitment to its corresponding organelle. Consistent with the present results, the a2 subunit of V-ATPase recruits ARNO, a GEF for the ARF6 GTPase, to early endosomal membranes in the renal epithelium, thereby supporting protein reabsorption in renal proximal tubules^32,33^. Taken together, these findings suggest that the a subunit isoforms of V-ATPase recruit GEFs and small GTPases to the corresponding organelles, thereby potentially determining the direction and timing of organelle trafficking, which is essential for establishing cell-specific functions. Further analysis is necessary to fully elucidate the mechanism underlying organelle trafficking regulated by V-ATPase a subunit isoforms.

## Methods

### Animals and cell culture

C57BL/6-a3 ± heterozygous mice (BRC no. 04421) were purchased from the RIKEN BioResource Center and mated to obtain homozygous a3+/+ wild-type (WT) and a3−/− knockout (a3KO) mice. All animal experiments complied with the Animal Experimental Guidelines of Iwate Medical University and the Act on Welfare and Management of Animals of Japan, and were approved by the Ethics Committee for Animal Research at Iwate Medical University (approval no. 28-017 and 03-019). The study was carried out in compliance with the ARRIVE guidelines. Isolation of macrophages and induction of osteoclast differentiation were performed as described previously^6,34^. Spleen cells derived from 2-week-old WT and a3KO mice were plated at a density of 0.3 × 10^6^ and 0.1 × 10^5^ cells/cm^2^, respectively. Cells were cultured in Minimum Essential Medium alpha (MEMα) supplemented with 10% fetal bovine serum, 100 U/mL penicillin, 100 µg/mL streptomycin, and 25 ng/mL macrophage-colony stimulating factor (R&D Systems) for 3 days, and macrophages were obtained as the adherent cells. Cells were further incubated in the presence of 200 ng/mL receptor activator of nuclear factor kappa B ligand (RANKL) (Oriental Yeast).

HEK293T and Plat-E cells were purchased from the RIKEN BioResource Center (RCB2202) and Cell Biolab, respectively, and cultured in Dulbecco’s modified Eagle’s medium containing 10% fetal bovine serum and antibiotics. Unless otherwise indicated, all reagents used for cell culture were from Thermo Scientific.

### Retrovirus infection

For retrovirus production, 1 × 10^6^ cells of Plat-E packaging cells (Cell Biolab) were plated in 60 mm dishes and were transfected with pMX retroviral vectors using XtremeGENE9 (Sigma-Aldrich). Spleen macrophages were infected with the retroviruses and selected with 2 µg/mL puromycin; then, they were replated in 24-well multi-well dishes at 1.0 × 10^4^ cells per well and cultured for a further 2 days in the absence of puromycin. Cells were differentiated into osteoclasts by culturing with 200 ng/mL RANKL^34,35^.

### Construction of plasmids

FLAG-tagged a3 and V5-tagged Rab7(T22N) in pcDNA3.1 were constructed as described previously^6^. To construct pcDNA3.1/V5-Mon1A and pcDNA3.1/V5-Ccz1, DNA fragments of Mon1A and Ccz1 were obtained by reverse transcriptase-polymerase chain reaction (RT-PCR) using total RNA isolated from RAW264.7 cells. DNA fragments encoding deletion mutants of a3, Mon1A, and Ccz1 were amplified by PCR using the respective plasmids containing wild-type cDNAs and primers. These fragments were digested by restriction enzymes (BamHI and NotI for a3 and Ccz1; BglII and NotI for Mon1A) and cloned into pcDNA3.1/FLAG and pcDNA3.1/V5 vectors, BamHI and NotI-digested pcDNA/FLAG-Rab7 and pcDNA3.1/V5-Rab7 plasmids. Mon1A mutations (G166P/K167D and N262W/D264A/R266A) resulting in decreased binding to Rab7 were introduced by oligonucleotide-directed site-specific mutagenesis PCR^12,14^. Primers used in this study are shown in Supplementary Table S1 online.

### Immunoprecipitation and western blotting

Immunoprecipitation was performed as described previously^6,34^. HEK293T cells (0.5 × 10^6^ cells per 60 mm dish) were transfected with the expression plasmids using XtremeGENE9. At 48-h post-transfection, cells were lysed in IP buffer (1% Triton X-100, 10% glycerol, 50 mM Tris–HCl pH 7.4, 150 mM NaCl, 1 mM dithiothreitol, 1 mM EDTA, 1 mM phenylmethanesulfonyl fluoride, and a protease inhibitor cocktail), and the lysates were used for immunoprecipitation with anti-FLAG antibody. Immunoprecipitates were analyzed by SDS–polyacrylamide gel electrophoresis and western blotting using an ECL Prime detection kit (GE Healthcare). Chemiluminescence was detected using the LAS-3000 imaging system (FUJIFILM). 15% SDS–polyacrylamide gels were used for Fig. 3c (FLAG), Fig. 4c (V5), and Fig. 5b,c (V5). For other western blot analyses, 10.5% SDS–polyacrylamide gels were used.

### Fluorescence microscopy

Immunostaining was performed as described previously^6,35^. Cells were fixed with 4% paraformaldehyde, permeabilized with P-sol (phosphate-buffered saline containing 0.4% saponin, 1% bovine serum albumin, and 2% normal goat serum), and incubated with primary antibodies in P-sol at 4 °C overnight, followed by fluorophore-conjugated secondary antibodies for 1 h at room temperature. Fluorescence images were acquired using an FV-1000 confocal microscope equipped with a 100 × objective, NA 1.40, and analyzed with FV10-ASW software (OLYMPUS).

### Antibodies

Information of antibodies can be found in Supplementary Table S2 online. Antibodies against FLAG, β-actin, and the B2 subunit of V-ATPase were purchased from Sigma-Aldrich. Antibodies to V5, Ccz1, CD68, and the A subunit of V-ATPase were obtained from Thermo Scientific, Santa Cruz, Hycult Biotech, and Abcam, respectively. Horseradish peroxidase (HRP)-conjugated anti-mouse and anti-rabbit IgGs were from GE Healthcare. Clean-Blot, an HRP-conjugated antibody for post-immunoprecipitation western blot detection of native primary antibodies, was purchased from Thermo Scientific. Alexa-conjugated secondary antibodies were from Thermo Scientific.

### Quantification and statistical analysis

The signal intensities of western blot bands were quantified using ImageJ. The co-localization of CD68 with Ccz1 was examined using FV10-ASW software for the confocal FV-1000 microscope. The F-test and unpaired two-tailed Student’s *t*-test were performed using Microsoft Excel software. P < 0.05 was considered statistically significant. All experiments were independently performed three times. Source data for Fig. 2c has been provided in Supplementary Table S3 online.

## Supplementary Information

Supplementary Information.

## Abbreviations

V-ATPase: Vacuolar-type proton-pumping ATPase
GEF: Guanine nucleotide exchange factor
WT: Wild-type
KO: Knockout
MEM: Minimum essential medium alpha
RANKL: Receptor activator of nuclear factor kappa B ligand
PCR: Polymerase chain reaction
HRP: Horseradish peroxidase
