# Supplementary material for: The lysosomal V-ATPase a3 subunit is involved in localization of Mon1-Ccz1, the GEF for Rab7, to secretory lysosomes in osteoclasts
Source: Sci Rep. 2022 May 19;12:8455. doi: 10.1038/s41598-022-12397-w (PMC9120031; doi:10.1038/s41598-022-12397-w)
Supplement: Supplementary file 1 — Supplementary Information. [file 41598_2022_12397_MOESM1_ESM.pdf]

The lysosomal V-ATPase a3 subunit is involved in localization of Mon1-Ccz1, the GEF for Rab7, to secretory lysosomes in osteoclasts

Naomi Matsumoto[1], Mizuki Sekiya[1], Ge-Hong Sun-Wada[2], Yoh Wada[3], Mayumi Nakanishi-Matsui[1,*]

[1]Division of Biochemistry, School of Pharmacy, Iwate Medical University, Yahaba, Iwate 028-3694, Japan

[2]Department of Biochemistry, Faculty of Pharmaceutical Sciences, Doshisha Women's College, Kyotanabe, Kyoto 610-0395, Japan

[3]Division of Biological Sciences, Institute of Scientific and Industrial Research, Osaka University, Ibaraki, Osaka 567-0047

a b

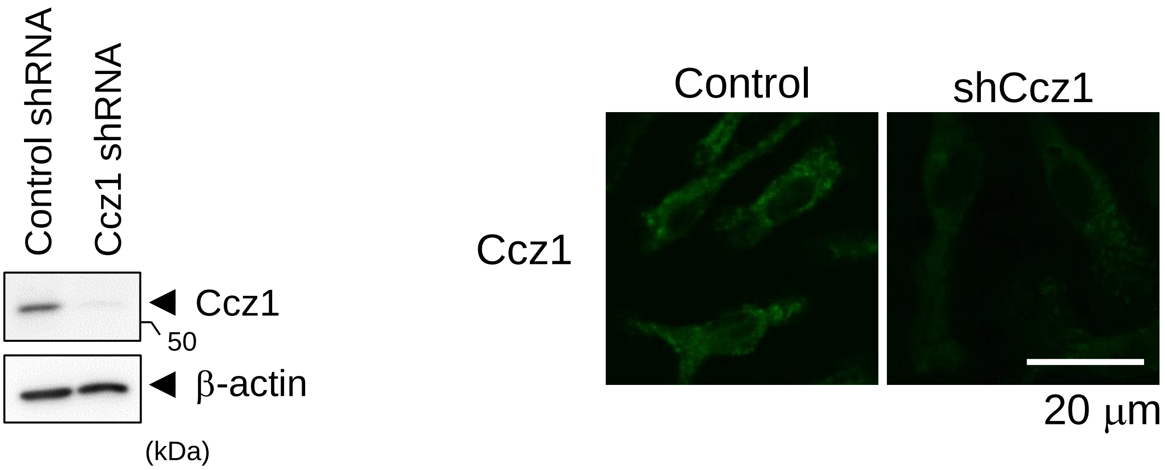

c

**Oligonucleotides for shRNA**	**sequences (5'-3')**
top	GATCCGCGTATGAAGAGCTCCATTTAATTCAAGAGATTAAATGGAGCTCTTCATACGCTTTTTTACGCGTG
bottom	AATTCACGCGTAAAAAAGCGTATGAAGAGCTCCATTTAATCTCTTGAATTAAATGGAGCTCTTCATACGCG

Supplementary Figure S1

Expression (**a**) and immunofluorescent staining (**b**) of Ccz1 in spleen macrophages treated with Ccz1 shRNA. (**c**) Sequence of oligonucleotides used for shRNA knockdown. These oligonucleotides were annealed and ligated into RNAi-Ready pSIREN-RetroQ vector (Clontech Laboratories). Retrovirus production and infection to spleen macrophages were done as described in Methods.

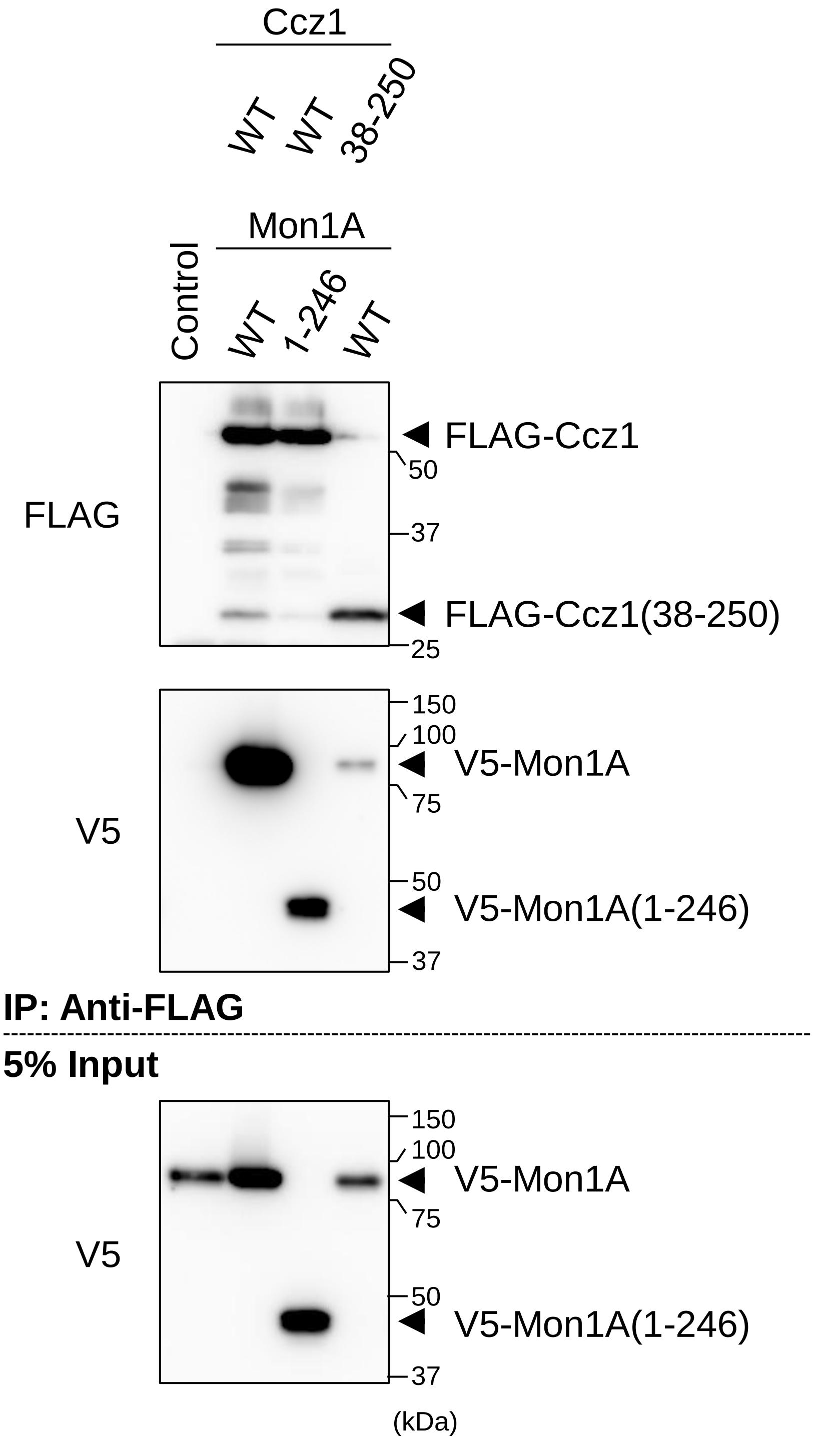

Supplementary Figure S2

Interaction between Mon1A and Ccz1 mutants exhibiting decreased interaction with a3N.

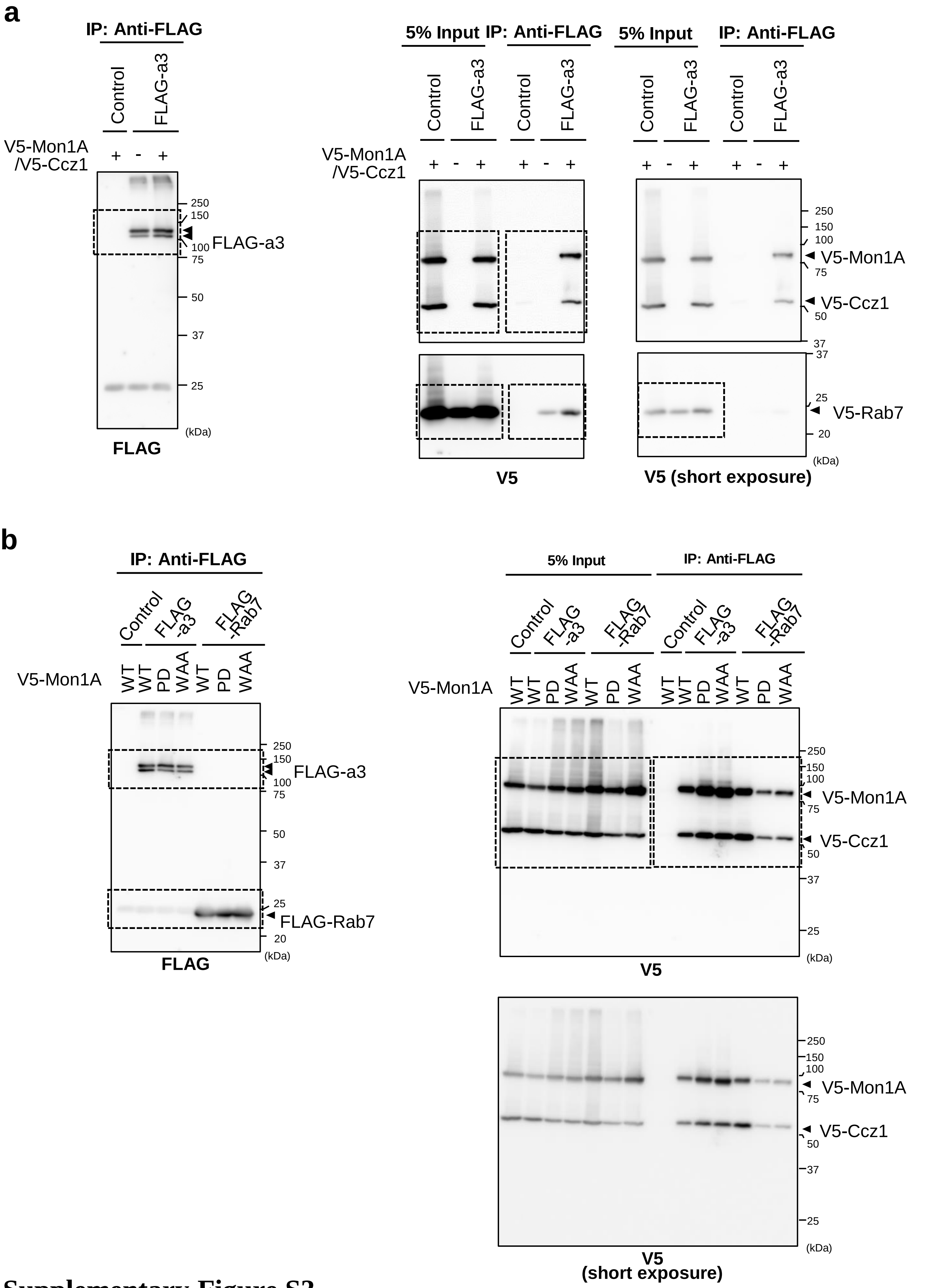

Supplementary Figure S3

a and **b** are unprocessed scans of Figures 1b and 1c, respectively. Short exposures of high-contrast of blots (V5) are also shown.

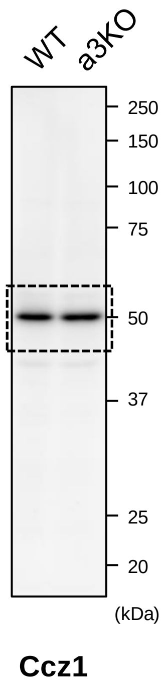

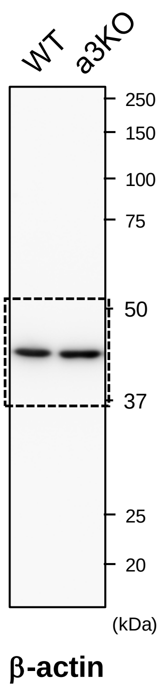

Supplementary Figure S4

Unprocessed scans of Figure 2a.

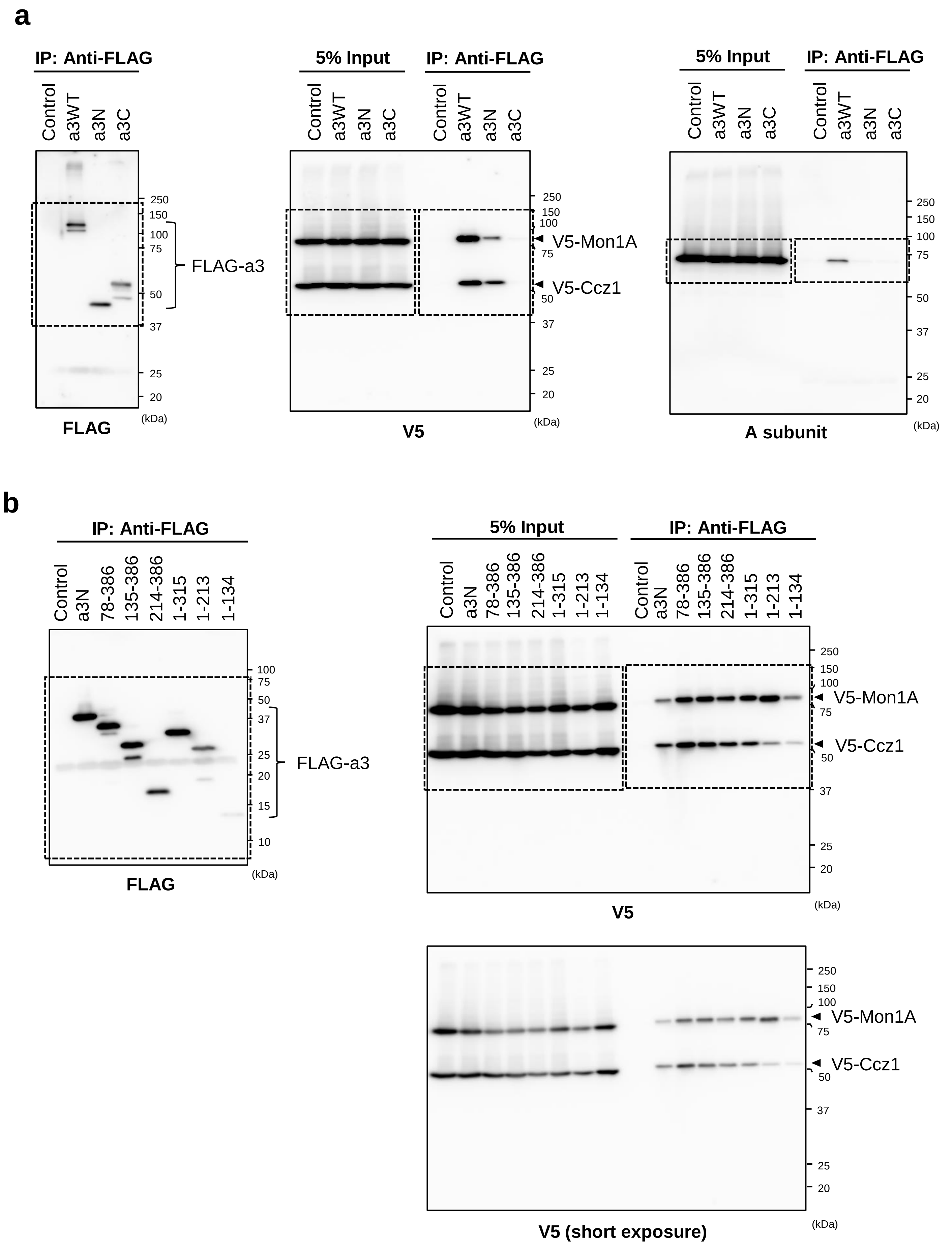

Supplementary Figure S5

a and **b** are unprocessed scans of Figures 3b and 3c, respectively. Short exposure of high-contrast of blot (Fig. S3b, V5) is also shown.

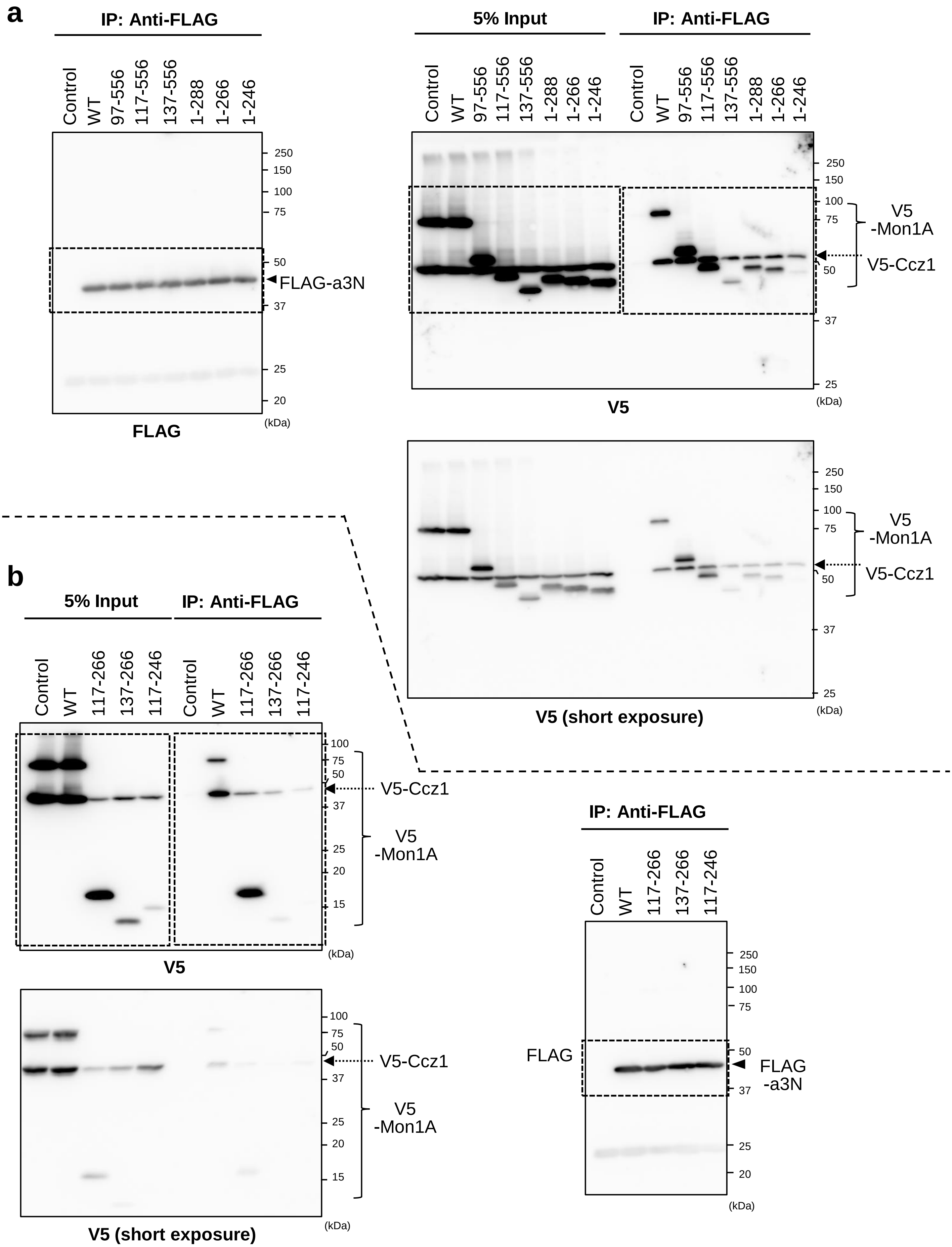

Supplementary Figure S6

a and **b** are unprocessed scans of Figures 4b and 4c, respectively. Short exposures of high-contrast of blots (V5) are also shown.

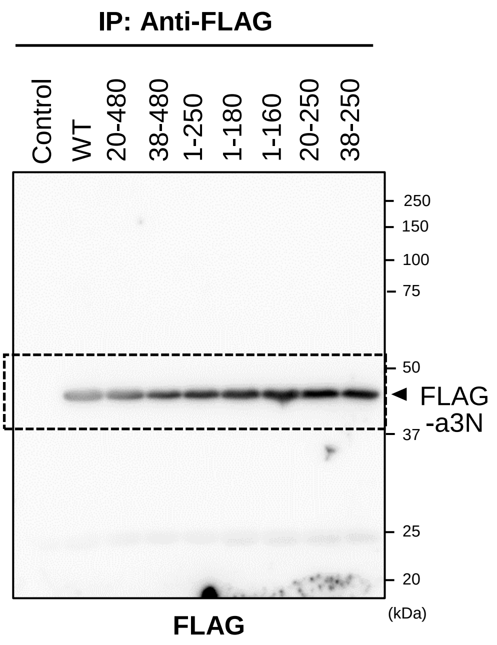

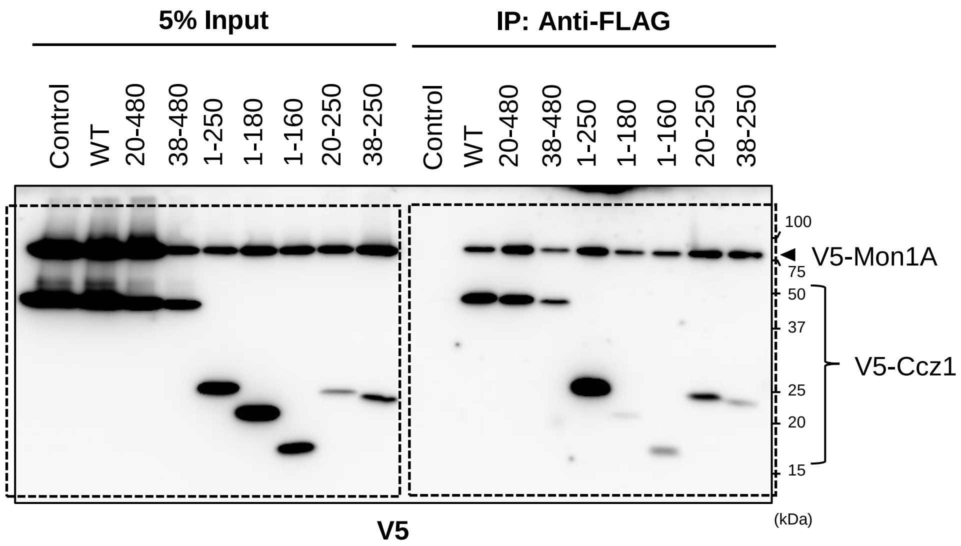

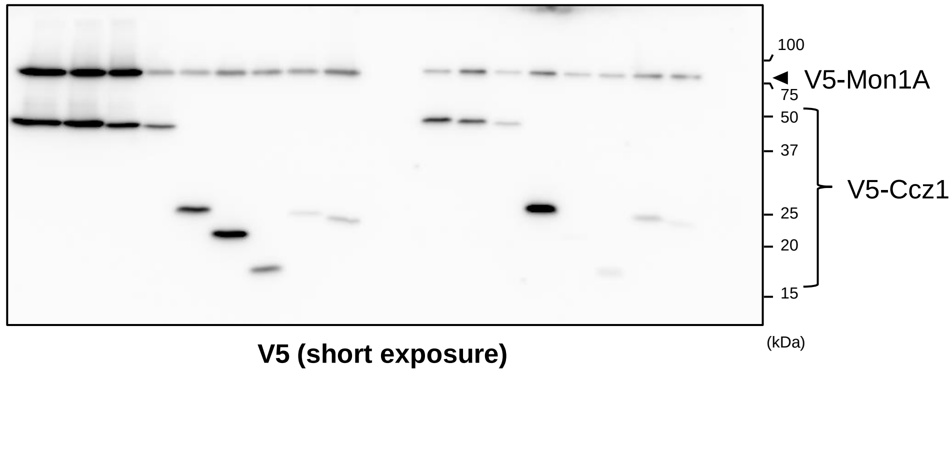

Supplementary Figure S7

Unprocessed scans of Figure 5b. Short exposures of high-contrast of blot (V5) is also shown.

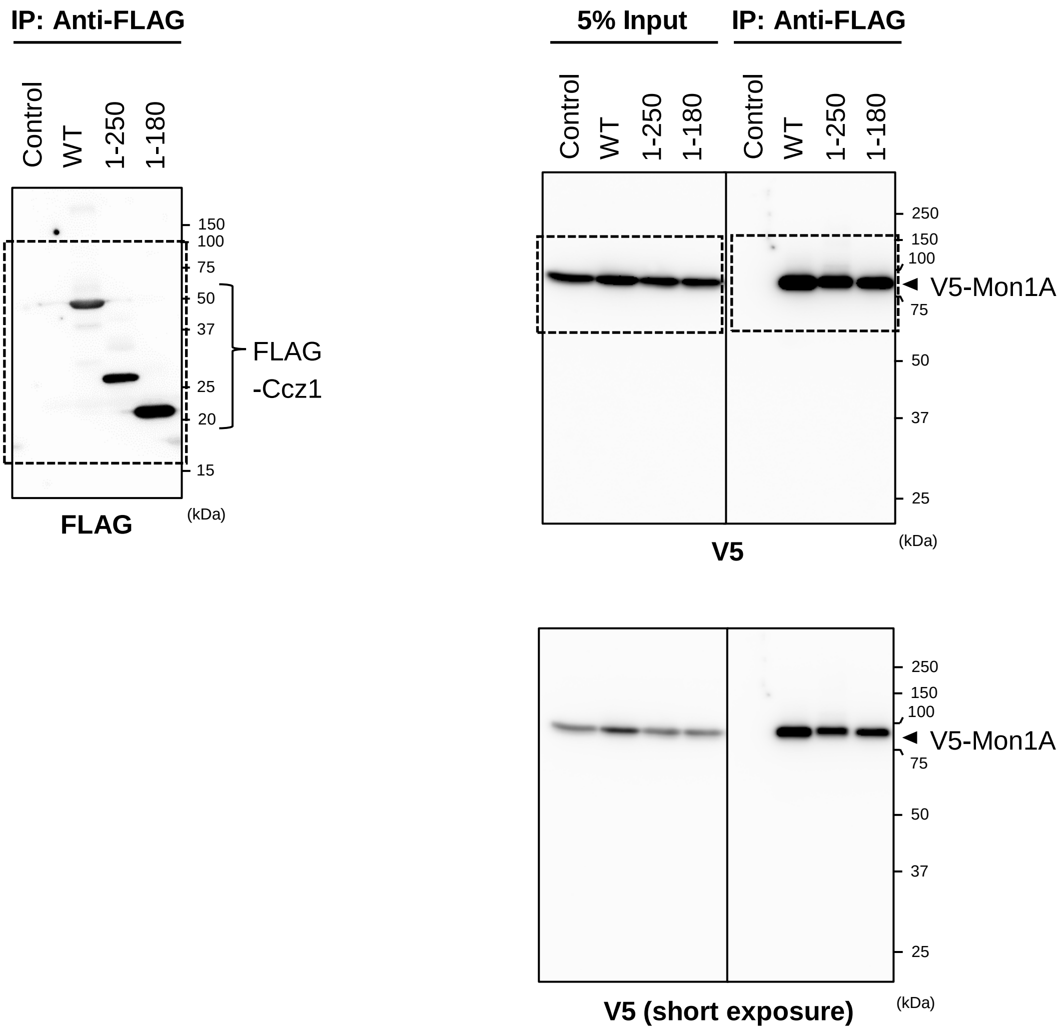

Supplementary Figure S8

Unprocessed scans of Figure 5c. Short exposure of high-contrast of blot (V5) is also shown. Right panels are grouping of blots cropped from different parts of the same gel.

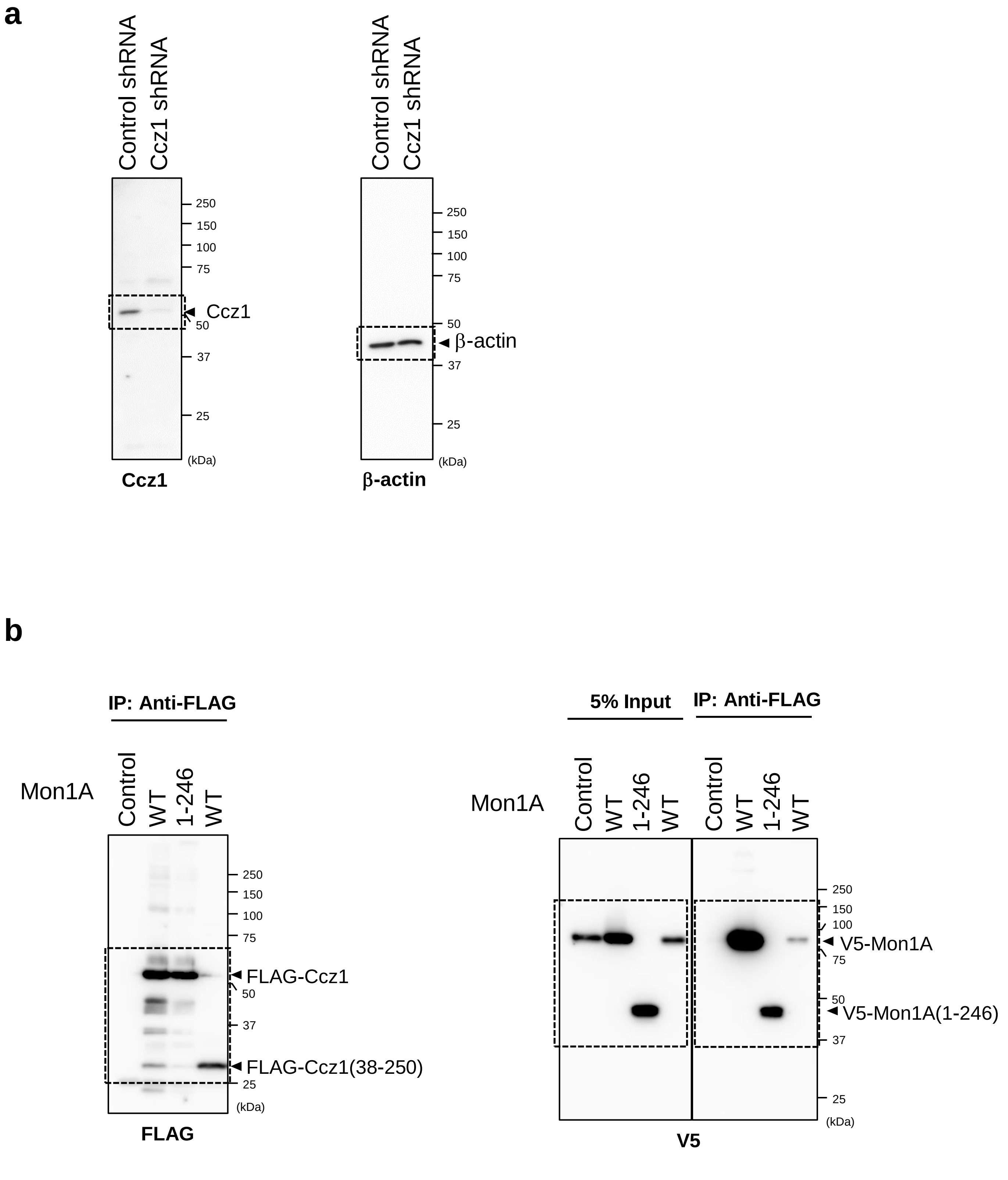

Supplementary Figure S9

a and **b** are unprocessed scans of Supplementary Figures S1a and S2, respectively.

Primers	sequences (5'-3')	italic
**a3**		
a3N_R(a3_386R)	AATT*GCGGCCGC*CTATTCCCTGTAGCGGCCCAC	NotI
a3C_F(a3_387F)	CGCTAC*GGATCC*GTTAACCCTGCTCCCTAC	BamHI
a3_78F	TGGGCA*GGATCC*ACGCTGGCCCCACCTGAG	BamHI
a3_135F	GTGCTG*GGATCC*AGCCACAGCCCGCCGGTG	BamHI
a3_214F	GCAACT*GGATCC*ACCTTTGTCATCTCCTAC	BamHI
a3_134R	TCAGTT*GCGGCCGC*CTACTGGCCCAGCACAGCCGA	NotI
a3_213R	TCAGAT*GCGGCCGC*CTACATCCAAGTTGCAGGCTC	NotI
a3_315R	AGCTCA*GCGGCCGC*CTATGTGGTGTTCACACTGCA	NotI
**Mon1a**		
Mon1a_F	CTGAAT*AGATCT*GCTGCTGACATGCAGAGGAAG	BglII
Mon1a_R	ATGGCA*GCGGCCGC*TCAATAGGTGAGGGGCGTGAG	NotI
M1a_(PD)F	AGTGAGGCACCTGACCCTGTGTATTCTCGCTAT	
M1a_(PD)R	ATACACAGGGTCAGGTGCCTCACTCAGTACAAA	
M1a_(WAA)F	CAGTGGTACGCCCTGGCTCGCCTGCTCTCGGGCTC	
M1a_(WAA)R	GCGAGCCAGGGCGTACCACTGCTTCTGCTGGAAGA	
M1a_97F	AGTCAA*AGATCT*CTAAGCACCCAGCTGACC	BglII
M1a_117F	AGTCAA*AGATCT*TCTGAAGACTGGCCGGAG	BglII
M1a_137F	AGTCAA*AGATCT*CAAGAAGGCTCAACTGAAG	BglII
M1a_288R	TAAGCT*GCGGCCGC*TCAACTGGGGTCTCGAGCCAT	NotI
M1a_266R	TAAGCT*GCGGCCGC*TCACCGCAGGTCGTAGTTCTG	NotI
M1a_246R	TAAGCT*GCGGCCGC*TCAGCTCAGGATCTGGTAGTA	NotI
**Ccz1**		
Ccz1_F	CTGAAT*GGATCC*GCGGCAGCCGCGGCCGGGCCGGGT	BamHI
Ccz1_R	ATGGCA*GCGGCCGC*TCAATCCAAGAAGAAGATGTTGTTGAA	NotI
Ccz_20F	AGTCAA*GGATCC*CCAGCGCTGCTGAGCTTC	BamHI
Ccz_38F	AGTCAA*GGATCC*GAGGAAGAAAATAAAATTCTG	BamHI
Ccz_250R	TAAGCT*GCGGCCGC*TCACAGTGAGGTGGTGAGGTA	NotI
Ccz_180R	AAGCT*GCGGCCGC*TCACTGCAAATGTAGTGTCTGC	NotI
Ccz_160R	TAAGCT*GCGGCCGC*TCACTTGACGCCTCCATCTTC	NotI

Supplementary Table S1.

Information on primers used for PCR.

Names	Clone	Origin	Catalog number	Company	Dilution	Assay
FLAG		rabbit polyclonal	F7425	SIGMA	1/1000	WB
V5		mouse monoclonal	R96025	Life Technologies	1/500	WB
Ccz1	B-7	mouse monoclonal	sc-514290	Santa Cruz Biotechnology	1/200, 1/50	WB, IF
β-actin	AC-15	mouse monoclonal	A5441	SIGMA	1/2000	WB
CD68	FA-11	rat monoclonal	HM1070	Hycult Biotechnology	1/200	IF
A subunit	EPR19270	rabbit monoclonal	ab199326	Abcam	1/500	WB
Clean Blot IP detection Reagent (HRP)			21230	Thermo SCIENTIFIC	1/1000	WB
Anti-Mouse IgG, HRP-Linked Whole Ab		sheep	NA931VS	GE Healthcare	1/1000	WB
Anti-Rabbit IgG, HRP-Linked Whole Ab		donkey	NA934VS	GE Healthcare	1/1000	WB
Alexa Fluor® 488 Goat Anti-Mouse IgG (H+L)		goat polyclonal	A11029	Life Technologies	1/200	IF
Alexa Fluor® 546 Goat Anti-Rat IgG (H+L)		goat polyclonal	A11081	Life Technologies	1/200	IF

Supplementary Table S2.
Information on antibodies used for this study.

	WT	a3KO
#1	36.26	3.72
#2	23.75	4.54
#3	47.43	5.06
#4	31.55	8.24
#5	36.71	2.29
#6	51.8	3.8
#7	33.66	5.83
#8	46.21	4.22
#9	18.25	2.28
#10	23.55	0.43
#11	20.96	2.1
#12	24.21	1.16
#13	36.7	0.58
#14	33.03	2.69
#15	25.93	0.3
av	32.667	3.149
SD	10.141	2.227
SE	2.618	0.575
F-test	0.000001173	
t-test	0.000000011	

Supplementary Table S3.

Statistics source data for Figure 2c. Co-localization of Ccz1 and CD68 was observed, and the ratios of CD68-positive pixels that were also Ccz1-positive were calculated.
